# Supplementary material for: Depression Earlier on in Life Predicts Frailty at 50 Years: Evidence from the 1958 British Birth Cohort Study
Source: J Clin Med. 2023 Aug 26;12(17):5568. doi: 10.3390/jcm12175568 (PMC10487987; doi:10.3390/jcm12175568)
Supplement: Supplementary file 1 [file jcm-12-05568-s001.zip › jcm-2556270-supplementary.pdf]

Supplementary File S1. Items used to create the frailty index

N	Description	Type
1	Limited: Mod activities-moving table, pushing vac, bowling, golf	Binary (Yes = 1 / No = 0)
2	Limited: Lifting or carrying groceries	Binary (Yes = 1 / No = 0)
3	Limited: Climbing several flights of stairs	Binary (Yes = 1 / No = 0)
4	Limited: Climbing one flight of stairs	Binary (Yes = 1 / No = 0)
5	Limited: Bending, kneeling or stooping	Binary (Yes = 1 / No = 0)
6	Limited: Walking 100 yards	Binary (Yes = 1 / No = 0)
7	Limited: Bathing or dressing yourself	Binary (Yes = 1 / No = 0)
8	How much bodily pain had during the past 4 weeks?	Likert (Very severe = 1 / Severe = 0.8 / Moderate = 0.6 / Mild = 0.4 / Very mild = 0.2 / None = 0)
9	How often woke and trouble refalling asleep in past 4wks	Likert (Very severe = 1 / Severe = 0.8 / Moderate = 0.6 / Mild = 0.4 / Very mild = 0.2 / None = 0)
10	BMI above or below healthy weight	BMI (<18.5 or ≥ 30 as =1 / 25 to <30 =0.5 / >=18.5 to <25 = 0)
11	Self-assessment of health compared to a year ago	Likert (Much worse = 1 / Worse = 0.75 / Same = 0.5 / Better = 0.25 / Much better = 0)
12	Self-assessment of health	Likert (Poor = 1 / Fair = 0.75 / Good = 0.5 / Very good = 0.25 / Excellent = 0)
13	Whether suffers health problem: Asthma or wheezy bronchitis	Binary (Yes = 1 / No = 0)
14	Whether suffers health problem: Diabetes	Binary (Yes = 1 / No = 0)
15	Whether suffers health problem: Convulsion, fit, epilepsy	Binary (Yes = 1 / No = 0)
16	Whether suffers health problem: Backache, prl disc ,sciatica	Binary (Yes = 1 / No = 0)
17	Whether suffers health problem: Cancer or leukaemia	Binary (Yes = 1 / No = 0)
18	Whether suffers health problem: Problems with hearing	Binary (Yes = 1 / No = 0)
19	Whether suffers health problem: Sight prob and glasses/lenses	Binary (Yes = 1 / No = 0)
20	Whether suffers health problem: High blood pressure	Binary (Yes = 1 / No = 0)
21	Whether suffers health problem: Migraine	Binary (Yes = 1 / No = 0)
22	Whether suffers health problem: Eczema/other skin problems	Binary (Yes = 1 / No = 0)
23	Whether suffers health problem: Chronic fatigue syn. (ME)	Binary (Yes = 1 / No = 0)
24	Whether suffers health problem: Stomach/bowels/gall bladder	Binary (Yes = 1 / No = 0)
25	Whether suffers health problem: Probs with bladder/ kidneys	Binary (Yes = 1 / No = 0)
26	Whether suffers health problem: Cough/bringing up phlegm	Binary (Yes = 1 / No = 0)
27	Cognitive test: Number of words correctly recalled	Quantiles (1st = 1 / 2nd = 0.75 / 3rd = 0.5 / 4th = 0.25 / 5th = 0)
28	Cognitive test: Number of animals mentioned	Quantiles (1st = 1 / 2nd = 0.75 / 3rd = 0.5 / 4th = 0.25 / 5th = 0)
29	Cognitive test: Number of cancellations correct	Quantiles (1st = 1 / 2nd = 0.75 / 3rd = 0.5 / 4th = 0.25 / 5th = 0)
30	Cognitive test: Number of words recalled after delay	Quantiles (1st = 1 / 2nd = 0.75 / 3rd = 0.5 / 4th = 0.25 / 5th = 0)
